# Sarcoidosis-Associated Pulmonary Hypertension

**DOI:** 10.3390/jcm13072054

**Published:** 2024-04-02

**Authors:** Dominique Israël-Biet, Jean Pastré, Hilario Nunes

**Affiliations:** 1 Université Paris Cité, 75006 Paris, France; 2 Service de Pneumologie, Hôpital Européen Georges Pompidou, AP-HP, 75015 Paris, France; jean.pastre@aphp.fr; 3 Service de Pneumologie, Hôpital Avivenne, AP-HP, 93000 Bobigny, France; hilario.nunes@aphp.fr; 4 Inserm UMR 1272 “Hypoxie et Poumon”, UFR de Santé, Médecine et Biologie Humaine (SMBH), Université Sorbonne Paris-Nord, 93000 Bobigny, France

**Keywords:** sarcoidosis, pulmonary hypertension, advanced pulmonary sarcoidosis complications

## Abstract

Sarcoidosis-associated pulmonary hypertension (SAPH) is a very severe complication of the disease, largely impacting its morbidity and being one of its strongest predictors of mortality. With the recent modifications of the hemodynamic definition of pulmonary hypertension (mean arterial pulmonary pressure >20 instead of <25 mmHg,) its prevalence is presently not precisely known, but it affects from 3 to 20% of sarcoid patients; mostly, although not exclusively, those with an advanced, fibrotic pulmonary disease. Its gold-standard diagnostic tool remains right heart catheterization (RHC). The decision to perform it relies on an expert decision after a non-invasive work-up, in which echocardiography remains the screening tool of choice. The mechanisms underlying SAPH, very often entangled, are crucial to define, as appropriate and personalized therapeutic strategies will aim at targeting the most significant ones. There are no recommendations so far as to the indications and modalities of the medical treatment of SAPH, which is based upon the opinion of a multidisciplinary team of sarcoidosis, pulmonary hypertension and sometimes lung transplant experts.

## 1. Introduction

Sarcoidosis-associated pulmonary hypertension (SAPH) is a very severe complication of sarcoidosis, strongly impacting its morbidity and being one of its strongest predictors of mortality [1,2,3,4,5,6]. Its prevalence is highly variable according to the populations studied and the diagnostic tool used. In addition, the definition of pulmonary hypertension has been recently modified [7] to a mean pulmonary artery pressure of 20 mmHg. Even when right heart catheterization (RHC) was used in previous studies to establish the diagnosis, this threshold was 25 mmHg and no prospective study has been published since then, precluding the possibility of present and reliable data about this prevalence. However, it is well known to be most frequent, although not exclusively, in advanced pulmonary sarcoidosis, and particularly in patients with end-stage disease on lung transplant waiting lists. Its physiopathology is complex, generally multifactorial and evolving during the course of the disease, and thus it is assigned to WHO Group 5 pulmonary hypertension (PH). Due to the very aspecific nature of its main clinical symptoms, mainly a persistent dyspnea frequently out of proportion with the parenchymal sarcoid involvement, systematic screening strategies should be established, particularly in cases with a recent decrease in DLCO ≤ 40% and/or a 6 mn walk distance ≤300 m. The definitive diagnosis requires right heart catheterization, the gold-standard diagnostic procedure. The decision to perform it in cases of clinical suspicion, possibly reinforced by imaging data, (CT scan, transthoracic echocardiography) has to be discussed within a multidisciplinary team comprising a sarcoidosis and a pulmonary hypertension expert. Considering its dark prognostic impact, the most appropriate treatment of SAPH if confirmed will be based upon the main pathogenic mechanisms underlying pulmonary hypertension, most often involving pulmonary vasculopathy or fibrotic processes. The optimal medical treatment of SAPH is not presently consensual, but in any case aims to specifically address the predominant mechanisms underlying SAPH in each individual patient. Establishing the most accurate SAPH phenotype is therefore crucial for appropriate and personalized management.

## 2. Epidemiology

The prevalence of SAPH remains difficult to evaluate due to the extreme heterogeneity of studies focusing on the topic. Averaging 3 to 8% in unselected populations of sarcoid patients, it has been reported (Table 1) with a range as wide as 2 to 74% [8]. This large heterogeneity is driven by three main explanations. Firstly, the large variety of methodologies reported partly accounts for this largely varying prevalence across the studies. Indeed, they include mostly retrospective studies but also some prospective ones, including registries, cross-sectional studies or studies based on the extraction of healthcare data. Secondly, the reported prevalence also varies with the type of sarcoidosis populations included in these studies. Indeed, the lowest prevalence is reported in studies focusing on general sarcoid populations, whereas that reported in patients with suggestive symptoms or signs of PH or with a more advanced disease is by far much higher. Thirdly, the prevalence also varies with the diagnostic tool used, with some studies using mostly transthoracic echocardiography (TTE), while others used right heart catheterization (RHC), the latter being the gold standard for diagnosis. Studies based on TTE usually report a higher prevalence of SAPH with a probable overestimation. 

One of the first studies to report SAPH prevalence is a prospective TTE-based Japanese one published in 2006 [11] which reported a prevalence of 5.7% in 212 patients. In this study, the diagnosis was based upon a right ventricle systolic pressure (RVSP) ≥ 40 mmHg. More recently, the PULSAR study (PULmonary hypertension in pulmonary SARcoidosis) [20], a large Dutch study investigating the PH prevalence in a predominantly Caucasian cohort of almost 400 consecutive sarcoid patients referred to a tertiary sarcoidosis center, used echocardiography and, if indicated, RHC. It reported a SAPH prevalence of 3%. These data are supported by the results of another study, which reported a prevalence of 2.9% in a smaller cohort from a German tertiary center [12]. Schimmelpennink et al. [21]. showed that the prevalence of SAPH in patients with a PF-ILD phenotype of advanced sarcoidosis, solely evaluated by the mean pulmonary artery diameter/ascending aorta diameter ratio, was more prevalent in progressive (24%) than in non-progressive (10%) fibrotic pulmonary sarcoidosis, but the difference was not significant. This diagnostic tool is in any case clearly not the recommended one. A recent meta-analysis by Zhang et al. that included 25 studies across the world demonstrated, despite the substantial heterogeneity of the studies, that SAPH prevalence widely varied with (1) the diagnostic method used, with RHC providing lower values than TTE; (2) the geographic origin of the patients; and (3) the type of sarcoid population studied. Indeed, SAPH was found in 16.4% of sarcoid patients when evaluated by TTE vs. 6.4% when RHC was used [22], supporting the requirement of using the gold standard, RHC, to establish a reliable diagnosis. The meta-analysis also showed that SAPH diagnosed with RHC reached a prevalence of 62.3% in patients with an advanced disease. 

Other retrospective studies based upon data extracted from National Healthcare diagnostic code databases are also available from the USA [17], Israël [18] and Germany [19], reporting a SAPH prevalence of 8.6%, 6.7% and 2.8%, respectively. Despite the very large numbers of patients included, these studies present a major limitation due to data (such as diagnosis) not being manually extracted and checked. 

Various parameters have been associated with a higher SAPH prevalence, such as age, female gender and mostly more advanced parenchymal lung disease. Indeed, stage 3 or 4 sarcoidosis patients have the highest prevalence of SAPH, which is as high as 73.8% in sarcoid patients referred for lung transplantation [10]. These epidemiological data should prompt clinicians to specifically look for SAPH in the most severe patients. Potential predisposing rare genetic variants have been recently reported in SAPH patients, indicating a possible implication of genetics in its development [23]. Finally, among the factors predisposing the development of SAPH, ethnic differences should be taken into account. Though most studies focused on Caucasian populations, Bourbonnais reported a prevalence of 14% in a prospective study of a large majority of African Americans [24], while Alhamad reported that of 20.8% in a cohort of 96 Arabic sarcoid patients [14]. 

Most importantly, the hemodynamic definition of PH was modified in 2022 [7], lowering the threshold of the mean PAP from 25 mmHg to 20 mmHg. No prospective study has been published since then on SAPH, and therefore data to identify the real prevalence of this very severe sarcoidosis complication are lacking and all the studies referred to were based upon the old definition of pulmonary hypertension. All the prevalence data published so far are bound, therefore, to be underestimated. Only prospective studies using the new hemodynamic definition of PH will be able to produce more accurate reports of the real prevalence of SAPH.

However, in 2021, S. Nathan reported a study aiming at analyzing the impact of the prevalence and outcome of PH in patients with COPD or IPF [25]. Not unexpectedly, the prevalence of precapillary PH was higher in both groups compared to that reported with the old definition. In the IPF group, the new definition might have performed slightly better than the old one in predicting outcome. Sarcoidosis was not included in the study and no data about SAPH prevalence can be derived from it.

Finally, three recent papers clearly summarize the important steps associated with the new PH definition [26,27,28]. They all highlight several crucial points: (1) the concept of mild PAH (mPAP between 20 and 25 mmHg, PVR between 2 and 3 WU), pointing to its value as an early indicator of increased risks of severe outcomes; (2) the negative impact of comorbidities on either cardio-vascular or respiratory outcome, with the individualization of a particular phenotype of patients with a decreased DLCO (<45% pred), usually affecting males and smokers with mild CT parenchymal lung abnormalities; and (3) the crucial step for the most appropriate individualized SAPH management, i.e., that of its most precise phenotyping, particularly its vasculopathic one.

Again, none of them specifically addressed SAPH, but rather iPAH and ILD-associated PH. All of these key observations might pave the way for very informative specific studies to come regarding sarcoidosis.

## 3. Classification and Pathogeny

**Figure 1:**
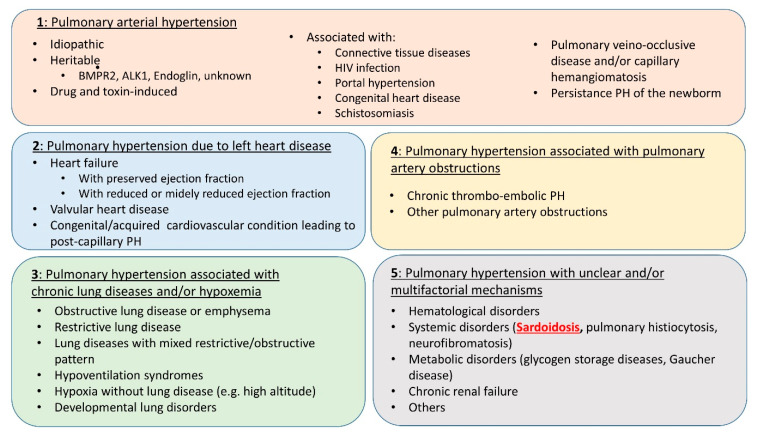
Clinical classification of pulmonary hypertension from [29].

**Figure 2:**
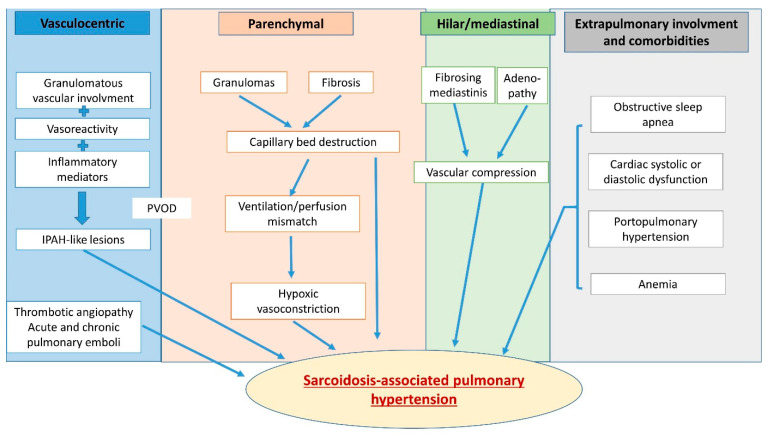
Multifactorial mechanisms lead to pulmonary hypertension in sarcoidosis and may include hypoxic vasoconstriction, pulmonary vascular rarefaction, parenchymal destruction, left heart disease with postcapillary PH, portal hypertension from liver disease, pulmonary vascular remodeling, changes resembling pulmonary veno-occlusive disease and extrinsic vascular compression due to fibrosing mediastinitis or enlarged lymph nodes.

One of the main difficulties of SAPH management is that it may be related to multiple and potentially entangled mechanisms. Considering this most often mixed pathogeny, the ESC/ERS Task Force and 6th World Symposium on Pulmonary Hypertension have placed SAPH in WHO group 5 [29] (Figure 1). Indeed, if SAPH is usually predominantly related to the underlying parenchymal lung disease, it may also involve mechanisms belonging to groups 1, 2, 3 and/or 4 (Figure 2). In any case, establishing the most precise cartography of the mechanisms underlying SAPH in each individual patient is absolutely key to the delineation of the subsequent therapeutic strategy. Therefore, despite the difficulty, the clinician should always, for the sake of the most appropriate individualized therapeutic management, aim at defining the predominant mechanism underlying SAPH. 

Two studies [23,30] showed that the expression of several genes clearly separates sarcoid patients with and without SAPH. The potential role of these particular genetic backgrounds in the development of SAPH is under investigation. 

As stated above, patients with more advanced parenchymal lung disease are more likely to develop PH, especially in cases of pulmonary fibrosis (stage 4). The pathophysiological mechanisms involved here are those observed in group 3 PH, such as capillary destruction due to parenchymal involvement and hypoxic pulmonary arterial vasoconstriction from ventilation/perfusion mismatches. This mechanism is the most frequent one in SAPH, with nearly 75% affected patients in a recent cohort of 40 subjects [31]. However, the degree of parenchymal alteration and functional restriction does not correlate with the severity of PH, and up to 20% of patients with SAPH do not have any radiographic evidence of parenchymal lung disease [9]. 

Among the other mechanisms potentially involved in sarcoidosis, vasculopathy may develop, mostly but not exclusively due to the granulomatous infiltration of pulmonary arterial and/or veinous walls. In this case, pathological changes as well as high levels of inflammatory mediators mimic alterations described in idiopathic pulmonary arterial hypertension (PAH, group 1) [32]. As in PAH, granulomatous vessel involvement may affect all the layers of the vasculature from the intima and media to the adventitia and smooth muscle. It may also affect the entirety of the pulmonary vascular tree [33] from the elastic arteries to the collecting venules, mimicking in this case a pulmonary veno-occlusive disease. In PAH, cigarette smoking contributes to the vasculopathy associated with endothelial dysfunction, apoptosis and remodeling, causing the “capillary drop-out” [26]. As sarcoid patients are mostly nonsmokers, this observation might not be relevant in SAPH. In contrast, a recent observation [34] details the mechanisms underlying sarcoid vasculopathy, particularly its venous component. It shows the presence of vascular granulomas adjacent to the perilymphatic ones and/or their vascular transmural spread. Even more interestingly, it shows the presence of numerous independent, unorganized intimal granulomas bulging into the vessels’ lumina, overlaid by endothelial cells with no thrombosis, whether or not a transmural granulomatous infiltration is observed. This might explain, at least in part, the development of pulmonary hypertension despite limited pulmonary involvement, for instance. Postcapillary PH, either alone or associated with precapillary PH, can also be observed in SAPH. Approximately 5–20% of patients, according to [35], develop cardiac sarcoidosis which might in turn lead to group 2 PH [36]. If conduction troubles and arrhythmias are the most frequent signs of cardiac sarcoidosis, systolic and even diastolic dysfunction can also occur during the course of the disease and lead to postcapillary PH. When both pre- and postcapillary PH are associated, RHC measurements will show a pulmonary artery wedge pressure (PAWP) > 15 mmHg in addition to a pulmonary vascular resistance >3 Wood’s units (WU). Chronic thromboembolism is also a potential mechanism of SAPH. Indeed, sarcoidosis is associated with a 2–3-fold risk of pulmonary embolism (PE) [37,38], and this association increases disease severity [39]. Sarcoid patients are therefore at increased risk of developing chronic thromboembolic pulmonary hypertension (CTEPH, group 4 PH) [40]. This should be systematically screened for because of the specific therapeutic management that ensues. 

Less frequently involved, several other mechanisms may underlie the development of SAPH, such as vascular compression by large mediastinal adenopathy [41] or fibrosis [42]. Liver sarcoidosis involvement might also be associated with porto-pulmonar hypertension [43]. Chronic anemia due to inflammation or to granulomatous bone marrow involvement can lead to high output heart failure and PH. Finally, obstructive sleep apnea syndrome (OSAS) with nocturnal hypoxemia, frequently observed in sarcoid patients, should be cited as a potentially worsening factor of a pre-existing PH [44]. 

Altogether, considering that SAPH may be due to various and frequently combined and evolving mechanisms, clinicians will frequently be very challenged about the exact phenotype in which to classify their patients. This step is, however, required for optimal management, and they should always try to dissect and weight the main drivers involved in each individual patient.

## 4. Diagnosis

SAPH strongly impacts morbidity, transplant-free survival and mortality of sarcoidosis [1,2,3]. Considering its dark prognosis; the requirement for a prompt referral to lung transplantation (LTx) centers for appropriate patients; the extreme difficulty in properly diagnosing the type of PH developed in sarcoid patients, usually very complex and multifactorial; and the therapeutical uncertainties associated with SAPH, these patients should benefit from a stepwise and comprehensive approach, non-invasive in the first step and then based upon RHC when appropriate. This diagnostic approach is the only valid one able to document the multidisciplinary discussions (expert centers with pulmonologists, cardiologists and PH experts) which will have to phenotype these patients [1,8] and to lead them to the most appropriate management.

The delay between the diagnosis of sarcoidosis and that of SAPH can be over a decade long, according to a French study [41] and a multi-national one [45]. If only less than 10% of patients can be asymptomatic, the diagnosis of SAPH can be largely delayed due to very aspecific symptoms such as chest pain, palpitations and/or cough. Only 8% of patients can be symptomatic. 

While some studies have long described several features (decreased pulmonary function and/or walk distance, DLCO < 60% pred, oxygen saturation <90% on 6MWT) as clinical predictors of PH in sarcoidosis [24], a recent one [46] used a multidisciplinary Delphi study to establish recommendations for screening strategies for PH in patients with interstitial lung diseases. The consensual triggers for PH suspicion were clinical signs and symptoms, chest CT and other imaging modalities, an abnormal pulse oxymetry, increase in serum BNP/NT-proBNP and worsening in pulmonary function tests or 6 min walk distance. Echocardiography and BNP/NT-proBNP were identified as relevant screening tools, while RHC was confirmed as the sole diagnostic proof. 

### 4.1. Symptoms and Signs

The most frequent symptom is an increasing and/or persistent dyspnea, sometimes out of proportion with the underlying parenchymal extent of the disease, with most patients presenting with a WHO functional class of III-IV and/or increase in supplemental oxygen needs [47,48,49]. Other clinical signs can include chest pain, light-headedness or even syncopes, and sometimes cough, none of these bearing any specificity. An increased P2 or S4 sound can also be noticed, while other signs of right ventricular dysfunction usually occur much later or in a rapidly evolutive disease.

### 4.2. ECG

Its utility as a screening tool for PH is uncertain and a normal result cannot rule out its presence [50]. A right axis deviation, a right ventricular hypertrophy or strain, and a right bundle block are usually signs of a late phase of the disease.

### 4.3. Pulmonary Function Tests (PFT)

No correlation exists between any of the spirometric nor plethysmographic values and the severity of PH, but a significant one does exist between the latter and a low DLCO < 60% pred [24,47,51,52]. SAPH is frequently associated with a decreased FVC and DLCO compared to sarcoid patients without PH [47,53]. However, as many as 28% of patients have a near-normal lung function [54]. Jose et al. showed in a cohort of 156 patients that a cutoff of % FVC < 60 pred and % DLCO < 50 pred reached a sensitivity of 62% and a negative predictive value of 85% for PH [55], confirming previous reports in the literature [9,24,56,57,58]. However, in the multidisciplinary Delphi study [46] cited above, the only PFT-related trigger for SAPH suspicion that reached a consensus were a % DLCO < 40% pred or rapidly declining (>15%), disproportionate to lung volumes (FVC/DLCO > 1.6). No consensus was reached on the use of FVC or TLC or any threshold for these parameters to be used as predictive factors. 

Decreased DLCO is a strong predictive marker of PH, as a % DLCO <60% pred indicates a 7-fold increase risk of PH [59,60] and as a reduced DLCO is among these parameters that with a consistent correlation with PH [10,57]. Finally, a significant correlation between BNP levels, 6-minute walk distance (6MWD), % DLCO and TTE-evaluated PASP was found in a small cohort of sarcoid patients [61]. 

In addition to being predictive for PH, both DLCO < 35% pred and a preserved FEV1/FVC were shown to be independent markers of outcome [45].

### 4.4. WMT

A decreased 6MWD < 350 m and a large desaturation (<90%) have long been reported to have a predictive value for SAPH in the literature [24]. A recent decrease in 6MWD should undoubtedly prompt a thorough evaluation for SAPH [46]. Of note is the fact that, in contrast, the absence of desaturation on exertion is strongly indicative of the absence of SAPH [59]. In Gupta’s study [62], a 6MWD < 300 m was the strongest predictor of mortality or LTx in SAPH, whereas no association with outcome was found with either a desaturation > 5%, an O_2_ saturation < 88% at the end of the test or a composite product of 6MWD and oxygen saturation. The 6MWD was significantly inversely correlated with sPAP. This held true for pre- as well as postcapillary PH. Although this is by no means specific to SAPH due to several potential confounding factors such as airway disease, fatigue and muscle involvement [62,63], the fact that in this study 6MWD correlated with Borg score as well as with FAS indicates that it is not only a strong predictor of PH severity in sarcoid patients but that it moreover seems to be able to capture the multifactorial effects of sarcoidosis on the 6MWT.

Again, in addition to being predictive for PH, a reduced 6MWD < 300m was associated with a reduced transplant-free survival [45]. 

### 4.5. Imaging

Multimodal imaging techniques in SAPH include chest X-ray, CT scan, ventilation/perfusion (V/Q) scanning, CMR and other innovative techniques [64].

#### 4.5.1. Chest X-ray 

Although most patients with SAPH have an advanced pulmonary fibrosis [9,41], it can develop whatever the Scadding stage. A retrospective study of 22 patients with SAPH showed that 32% of them had no fibrosis at the time of PH diagnosis (6). In a recent study, only half of the patients in a large cohort of SAPH were at stage 4, with no correlation between stages and mPAP, in line with the 66% stage 4 value in a more recent one [45]. In contrast, stage 1 was very rare (2%), supporting the idea that a quite normal parenchyma in sarcoid patients might indicate the absence of SAPH. 

#### 4.5.2. Thoracic CT Scan

Patients with SAPH are more likely to have a certain extent of pulmonary fibrosis compared to sarcoid patients without, leading to the recommendation of looking for SAPH in all sarcoid patients with fibrosis [53]. However, some patients with no consistent parenchymal abnormalities develop seemingly out-of-proportion PH [65]. A prospective evaluation of a large cohort of patients (*n* = 246) showed no difference on CT scan between patients with or without PH in terms of lymph node enlargement, parenchymal involvement and thickening of bronchovascular bundles [11]. In contrast to parenchymal abnormalities, some vascular images are good indicators of PH, such as a pulmonary artery diameter > 29 mm, a right ventricle (RV)-to-left ventricle (LV) ratio > 1, or a pulmonary artery (PA)/ascending aorta (AA) > 1 [53,64,65], with a 65% sensitivity and a 83% specificity for the latter [66]. The ratio of PA diameter to BSA is even more predictive [67]. 

A recent study [68] combining PET scanning and CMR showed a clear 18F-FDG uptake in the PA wall in 33 subjects with suspected cardiac sarcoidosis. Very interestingly, it also showed that in those undergoing an RHC, the mean PAP pressure was higher in those with a 18F-FDG uptake compared to those without (*p* = 0.003). SUV max in the PA wall correlated with PA pressure derived from RHC and/or TTE. In summary, 18F-FDG uptake in PA wall is associated with PH with an intensity correlating with the mean PAP. 

Given the well-known risk of VTE episodes and of CTEPH in sarcoidosis [40,69,70], a systematic search for the latter should be performed in sarcoid patients with a suspected PH. Pulmonary angiography will help to reach this diagnosis, but dual-energy CT (DECT) [64,71,72] provides more information, including morphological information on the vasculature and functional information on perfusion. Primarily used to replace V/Q scanning, which does not allow any evaluation of the lung parenchyma nor mediastinal lymph nodes, it has also been investigated as a screening tool for pulmonary hypertension whatever its cause [73]. Its value in the clinical work-up in this context, and particularly in SAPH, is still undetermined.

Finally, a loss of small pulmonary vessels on quantitative CT might indicate severe PH [74], but this has not been evaluated in the context of sarcoidosis.

#### 4.5.3. Cardiac MRI

Non-compulsory in the diagnostic approach of SAPH, it has proved to be of some help in other ILD-associated severe PH [75]. 

#### 4.5.4. TTE

The most important non-invasive tool to diagnose PH [50,76] it is highly recommended in patients with the above cited clinical symptoms and signs, reduced 6MWD, desaturation on exercise, reduced DLCO and PA/AA > 1 on CT scan. 

A multi-national study based on ReSAPH, PULSAR and the Cincinnati Sarcoid Clinic database evaluated 124 patients with an RHC-confirmed SAPH [77]. It showed a strong correlation between right ventricular systolic pressure (RVSP) and pulmonary artery systolic pressure (PASP) in patients with a FVC > 60% pred, less significant in those with a FVC < 50% pred. TTE estimation was inaccurate in as many as 51% of the patients, with an underestimation in those with a severe PH and an overestimation mostly in those without PH. 

Tricuspid regurgitant velocity (TRV) value, when measurable, is crucial to the evaluation of PH likeliness [78]: it rules out PH when < 2.9 m/s and confirms it when > 3.4 m/s. Other TTE indicators of probable PH are the analysis of both ventricles, pulmonary arteries, inferior vena cava and right atrium. An echocardiographic score including right atrial area, left ventricular eccentricity index and right-to-left ventricle ratio has been shown to be an accurate indicator of PH whatever the presence of a measurable TRV [76]. 

The ESC/ERS PH guidelines [7] have proposed an algorithm taking into account TVR and other indirect measures to classify patients with a high, intermediate and low probability of PH. In any case, and particularly for those with an intermediate TRV value and/or a low probability of PH, an expert team is required to define on a case-by-case basis which patients should undergo an RHC. For the expert authors of the WASOG statement on diagnosis and management of SAPH [1], factors influencing the decision to perform an RHC included evidence of RV dysfunction on TTE, a FVC < 50% pred, a decreased 6MWD and increased levels of BNP-NT-proBNP. They found no consensus for an RHC indication in patients with an intermediate probability of PH and severe parenchymal disease. However, TTE and RHC are compulsory in the pre-LTx evaluation. 

TTE can be difficult to perform and interpret due to the extent of parenchymal lung disease. Three-dimensional TTE has been shown to better evaluate RV function and regional abnormalities in other conditions, with a good predictive value of the outcome and mortality and a good correlation with cardiac magnetic resonance-derived RV ejection fraction [79,80,81,82]. These data have not been evaluated yet in SAPH.

#### 4.5.5. RHC

The gold standard for the diagnosis of PH (1), it should be performed in an expert PH center and its results discussed within a multidisciplinary team comprising PH, sarcoidosis and imaging experts. Compulsory in patients listed for LTx, it is otherwise discussed on a case-by-case basis, particularly taking into account the likelihood of PH established on the above parameters and the effects on therapeutical and management decisions. 

A cut-off mPAP value of >20 mmHg has been established in the recent guidelines for the diagnosis and treatment of PH [7], replacing that of >25 mmHg which had prevailed for decades. 

RHC provides a very consistent diagnosis of PH-ILD in cases of precapillary PH (mPAP ≥ 20 mmHg, PVR > 2 WU, PCWP ≤ 15 mmHg) with evidence of ILD on imaging [83]. 

Precapillary PH due to vascular disease is robustly defined by a PVR ≥ 3 WU, but remains likely if between 2 and 3 [1].

As far as the definition of severe SAPH goes, with the foreseeable impact of therapeutic measures and timing on LTx evaluation, one study showed that both mPAP ≥ 40 mmHg and PVR ≥ 5 WU were strongly associated with a shorter transplant-free survival and increased risk of death or LTx [84]. Interestingly, and in contrast to common definitions of severe PH in chronic lung diseases, neither a mPAP > 35 mmHg nor mPAP > 25 mmHg with cardiac index ≤ 2 L/min/m^2^ were associated with these outcomes. 

As pre- and postcapillary PH can coexist in SAPH, provocative maneuvers such as fluid challenge or exercise [1] might be necessary to definitely characterize them. 

#### 4.5.6. Biomarkers

BNP or NT-proBNP, good predictors of RV overload and worse outcome, are often increased in SAPH but with low sensitivity and specificity. 

## 5. Phenotypes

After an extensive but again case-by-case diagnostic approach, the most precise characterization of the mechanisms underlying SAPH in individual patients is desirable for the sake of the most appropriate and specific therapeutic management in the era of personalized medicine. It should, however, be highlighted that SAPH phenotyping is not a straightforward process but rather a dynamic one with multiple and evolving phenotypes during the course of the disease. 

Several recent studies have addressed SAPH phenotyping [31,85] aiming at establishing the predominant pathomechanisms in their cohorts: parenchymal lung disease, extrinsic compression of pulmonary vessels, pulmonary angiitis and microangiopathy (defined by Mathijssen as a precapillary PH with PVR > 3 WU with no or mild parenchymal disease and after exclusion of all other causes of PH), LV dysfunction and portal hypertension. 

In the precapillary PH groups, Mathijssen [31] showed that 6 of 37 patients were classified as having compression of pulmonary vasculature (4 due to fibrotic disease and 2 due to active sarcoidosis), 29 as parenchymal, 1 as suspected vasculopathy and 1 as CTEPH.

In a number of cases, the development of SAPH is unpredictable from patients’ presentation at diagnosis. Even though it is known to mainly affect those with advanced pulmonary sarcoidosis, with 65 to 80% of sarcoid patients with precapillary PH having stage 4 disease [31,41,47,48,57], fibrosis is not necessary for PH development. Surprisingly, no correlation has been found between mPAP or PVR and any spirometric or plethysmographic features. Patients with comparable radiological and functional presentations displayed very different PH severity [3,10,13]. Whether this might be partly related to genetic predispositions is under study [23].

## 6. Treatment

**Figure 3:**
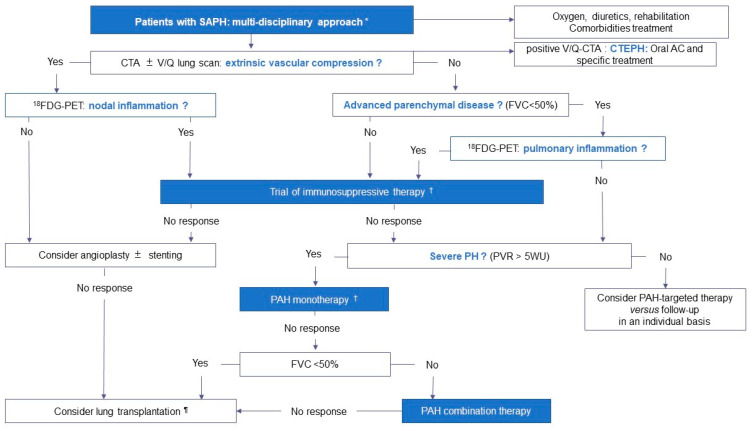
Proposed therapeutic approach of SAPH based on the mechanisms and phenotypes involved. * The therapeutic approach should be multidisciplinary, involving a sarcoidosis and a PH expert, and take into account the mechanisms involved in the development of PH, the severity of PH and the severity of the underlying parenchymal lung disease. ^†^ Anti-inflammatory treatment can be initiated before PAH-targeted therapy or in parallel. ^¶^ Referral for lung transplantation should not be delayed. Abbreviations: SAPH: sarcoidosis-associated pulmonary hypertension, CTA: computed tomography angiography, V/Q: ventilation/perfusion, AC: anticoagulant, CTEPH: chronic thromboembolic pulmonary hypertension, FVC: forced vital capacity, 18FDG-PET: 18F-2-fluoro-2-deoxy-D-glucose positron emission tomography, PVR: pulmonary vascular resistance, PAH: pulmonary arterial hypertension.

The optimal treatment of SAPH is not clearly established because of the limited number of well-designed studies [1,8]. Decisions should be made on a case-by-case basis and patients should be managed by an experienced multidisciplinary team with at least a sarcoidosis and PH expert [1,8]. The therapeutic approach depends primarily on the dominant pathophysiologic phenotype of SAPH, as illustrated in Figure 3. Supportive therapy remains the cornerstone of treatment, including supplemental oxygen in patients with resting and exertional hypoxemia, diuretics as needed and pulmonary rehabilitation to address possible deconditioning [1,8]. In addition, identification and appropriate treatment of comorbidities is critical, including OSAS, left heart dysfunction, acute or chronic thromboembolic disease, anemia and iron deficiency [1,8]. 

### 6.1. Obstructive Pulmonary Vasculopathy 

The first step of management is the identification and treatment of extrinsic vascular compression [1,8]. In fact, anti-inflammatory therapy can lead to a reduction in lymph node size and relief of vascular compression, which may be predicted by ^18^FDG-PET scans [41]. In the French registry, of the five patients with obstructive PH treated with anti-inflammatory therapy, two with metabolically active lymph nodes had a response at 6 months, but none of the three with mediastinal fibrosis did [41]. 

In highly selective cases with mediastinal fibrosis and proximal PA stenosis, angioplasty with or without stent placement may be beneficial, even though endovascular procedures are associated with high morbidity [86,87]. In a prospective Chinese series, eight patients with SAPH and PA stenosis failing to respond to 2 months of prednisone underwent interventional therapy (balloon angioplasty in all cases plus stenting in five) and exhibited a dramatic improvement in hemodynamics (decrease in mPAP from 42.5 ± 4.6 to 20.5 ± 3.2 mmHg, *p* = 0.035, and PVR from 12.3 ± 1.2 to 3.8 ± 0.3 WU, *p* = 0.004) and in 6MWD (increase from 236.8 ± 36.7 to 456.4 ± 48.2 m, *p* = 0.028) at 3 months [86]. One patient developed tachycardia, one thromboembolism, one hemoptysis and one PA dissection [86].

### 6.2. Treatment of Parenchymal Lung Disease

Despite little available data, it makes intuitive sense to control inflammation either before or in parallel to PH treatment in patients with SAPH and active parenchymal granulomas [1,8]. In an early series including 24 patients with pulmonary sarcoidosis, of whom 3 showed PH at rest and 18 showed PH on exercising, treated with 12 months of corticosteroids, 92% showed improvements on chest radiography and PFTs, but only half demonstrated improved hemodynamics [88]. In another study on 10 patients with SAPH, 3–6 months of corticosteroids resulted in a sustained amelioration of hemodynamics in 3/5 cases without pulmonary fibrosis, but no change in those 5 with stage IV [57]. Among the six patients with severe SAPH and parenchymal lung disease who had immunosuppressive therapy alone in the French registry, two with stage IV improved in terms of hemodynamics at 6 months, but not in terms of NYHA functional class or 6MWD [41]. ^18^FDG-PET scan may be particularly useful for gauging residual activity in patients with SAPH and fibrotic pulmonary disease and guide decisions regarding the initiation or escalation of immunosuppressive therapy [1,8].

### 6.3. Treatment of Vascular Disease and Use of PAH Agents

There are four main classes of drugs accepted for PAH therapy [7]: (1) calcium channel blockers, which are reserved for patients with a positive acute vasodilator response but are not indicated in group 5 PH, (2) endothelin-1 receptor antagonists (ERA) (including Bosentan and Ambrisentan), (3) phosphodiesterase-5 inhibitors (PDE5-i) (including Sildenafil and Tadalafil) and guanylate cyclase stimulators (including Riociguat), and (4) prostacyclin analogues (including inhaled Iloprost, inhaled Treprostinil and intravenous epoprostenol) and prostacyclin receptor agonists (including Selexipag). 

In SAPH, the use of therapy directed against vascular disease is still a matter of debate [1,8]. On the one hand, the possible role of dominant vasculopathy makes this therapeutic option appealing [1,8]. On the other, there is some concern over systemic pulmonary vasodilators in SAPH. First, they may lead to hypoxemia worsening in patients with parenchymal lung disease, because of the inhibition of hypoxic pulmonary vasoconstriction with subsequent increased ventilation/perfusion mismatch and shunting [1,8]. Second, the venous component that exists in a subset of patients may be at risk of drug-induced pulmonary edema [1,8].

In the French registry, 97/126 (77%) patients with severe SAPH received PAH-targeted therapy, including 86% with monotherapy (ERA: n = 60, PDE5-i: n = 20, intravenous Epoprostenol: n = 2, inhaled Iloprost: n = 1) and 14% with combination therapy (ERA + PDE5-i: n = 12, ERA + prostanoid: n = 2) [41]. In the international registry including unselected patients with SAPH, 115/159 (72.3%) received PAH-targeted therapy, which consisted of monotherapy in 88.2% (PDE5-i: n = 86, ERA: n = 56), bitherapy in 17.6% (PDE5-i + ERA: n = 28), and tritherapy in 6.3% (PDE5-I + ERA + prostanoid: n = 10) [45]. In both registries, PAH-targeted therapy was not associated with decreased mortality, provided that treated patients had a significantly worse condition at baseline than the untreated ones [41,45,84].

The available data on the long-term efficacy and safety of PAH-targeted therapy in SAPH are scarce and results are conflicting [1,8]. The main studies are summarized in Table 2. Most studies are retrospective small series that report diverse PAH regimens and do not take into account the variability of SAPH phenotypes [1,8]. To date, there are only three prospective uncontrolled open-label trials on inhaled iloprost [89], Ambrisentan [90] and Tadalafil [91], and two double-blind randomized placebo-controlled trials (RPCTs) on Bosentan [92] and Riociguat [93]. 

PAH therapy is generally beneficial in SAPH in terms of hemodynamics, but this effect is inconsistently accompanied by improvements in exercise capacity, quality of life (QOL) or BNP [1,8]. The Bosentan RPCT included 39 patients with no restrictions upon the severity of PH or functional alteration (Bosentan: n = 25, placebo: n = 14) [92]. Twenty-three patients completed 16 weeks of Bosentan and showed improvement in mPAP (decrease of 4 ± 6.6 mm Hg, *p* = 0.0105) and PVR (decrease of 1.7 ± 2.75 WU, *p* = 0.0104), whereas there was no change in hemodynamics with placebo. No significant change was observed in 6MWD or QOL [92]. Changes in hemodynamics and 6MWD on Bosentan did not differ according to FVC > or ≤50% [92]. The proportion of patients with worsening desaturation was similar between Bosentan and placebo [92].

The Riociguat RPCT included 16 patients with SAPH and FVC > 50% (Riociguat: n = 8, placebo: n= 8) [93]. After 1 year, treated patients demonstrated a significantly delayed time to clinical worsening compared to placebo [93]. The Riociguat group had an improvement in 6MWTD of +42.7 m (*p* < 0.025), whereas the placebo group had a decline of 55.9 m. No significant change was observed in QOL [93]. In addition, no worsening in oxygenation was noted under Riociguat [93].

In the French registry repeat assessments were performed after a median period of 4.5 months in 81/97 patients initiated on PAH-targeted therapy [41]. There were significant improvements in all hemodynamic variables (mPAP fell from 48 ± 9 to 42 ± 11 mmHg, *p* < 0.00001 and PVR from 9.7 ± 4.4 to 6.9 ± 3.0 WU, *p* < 0.00001). There was also an improvement in NYHA functional class but no significant change in 6MWD (324 ± 138 versus 311 ± 127, *p* = 0.33) [41]. Interestingly, in contrast to a previous study suggesting a better effect of PAH therapy in patients with more preserved FVC [95], no difference was found in both 6MWTD and hemodynamics on treatment according to the presence of stage IV disease or severity of restrictive physiology (FVC > or ≤50%) [41]. 

To summarize, off-label use of PAH drugs should be considered on an individualized basis in a expert PH center after taking into account the mechanisms involved in the development of PH, the severity of PH and the severity of the underlying parenchymal lung disease (Figure 3) [1,8]. There is no definite advantage of one drug over the others in SAPH [1,8]. In patients with a predominant parenchymal lung disease phenotype, PDE5-i is usually preferred [7]. Experts should facilitate the entry of patients into RPCT. After the positive results of INCREASE trial [99], inhaled treprostinil has been approved in the United States for treating ILD-PH and it is currently being studied in SAPH in the SAPPHIRE RPCT (NCT03814317). The SPHINX RPCT on Selexipag has been stopped after the enrollment of 10 patients (NCT03942211). Even though the hazard of PAH therapy seems marginal, it is prudent to monitor gas exchanges in patients with parenchymal lung disease [1,8].

### 6.4. Transplantation

Given the high mortality rate of SAPH, lung or heart–lung transplantation should be considered in otherwise eligible candidates [1,8]. However, the difficulty of prognosticating survival is a major factor confounding the issue of timing of referral in SAPH. Patients with SAPH have a greater likelihood of succumbing while on the waiting list [100], suggesting that referral tends to occur too late. In patients with advanced pulmonary sarcoidosis, the presence of PH should prompt referral for lung transplantation [1,8]. In case of predominant vascular phenotypes, it seems reasonable to refer patients who have failed to respond to PAH-targeted therapy [1,8]. Post-transplant survival in sarcoidosis patients is similar to that of other indications, and SAPH does not seem to be associated with higher mortality after lung transplantation [101].

## 7. Outcomes

As an independent risk factor for mortality [1,3,41,45,50,102], SAPH carries a 10-fold increased risk of death [24,56,103]. The predictors of adverse outcomes in SAPH are WHO functional class IV, RV dysfunction, severe lung fibrosis, 6MWD < 300m, DLCO <35% pred, and persistent increase in NT-proBNP after 3–9 months of vasodilators [45,49,62].

In a recent study of predictors of mortality on the LTx waitlist for sarcoidosis, severe PH was the most significant one [103], whereas a previous study [100], carried out in a single center, identified other markers (including DLCO and composite physiological index), but not PH, as predictors of death on the waiting list [100].

In addition to its strong impact on mortality, SAPH also increases morbidity with increased supplemental oxygen requirements [41,47], healthcare resource utilization [104], burden of functional capacity [48,49,100] and need for caregiver assistance. It also negatively impacts quality of life and the employment status. 

Finally, a recent study has addressed the question of risk factors for hospitalization in patients with SAPH undergoing LTx evaluation [105], as it has been addressed in group 1 PH [106,107]. It showed that 60% of sarcoid patients with PH were hospitalized at least once for respiratory failure before LTx or death. Treatment with vasodilators was significantly associated with a 80% decrease in risk of hospitalization [105]. This important finding might be taken into account in therapeutic decision making for SAPH patients, in whom guidelines are presently poorly validated. 

## 8. Conclusions

SAPH is a very severe complication of sarcoidosis, resulting from complex and often entangled mechanisms. Its diagnosis is often very challenging due to the poor specificity of its warning signs. These should lead to a thorough non-invasive work-up and, to confirm the diagnosis after a multidisciplinary discussion, to right heart catheterization. Only the most precise evaluation of the underlying mechanisms involved will allow proper therapeutic management. A part of the medical treatment will specifically address the vascular component of the disease, but the proper vasodilators to be used are not presently consensual. Ongoing properly designed clinical trials will largely help to define strong recommendations of the subject. In any case, this strategy will have to be discussed, repeatedly if necessary, with an expert team of sarcoidosis, pulmonary hypertension and at times lung transplant experts.

## Figures and Tables

**Table 1:** Studies reporting the prevalence of sarcoidosis-associated pulmonary hypertension and their characteristics.

Study	Year	N	Country	Ethnicity	Study Design	Diagnostic Method	Pre-Capillary PH by RHC	Prevalence	Comment
Sulica et al. [9]	2005	106	USA	NA	Retrospective	TTE	NA	51	48% of patients with stage 4
Shorr et al. [10]	2005	363	USA	A-A (71.6%)	Retrospective	RHC	NA	73.8	Population of sarcoidosis listed for lung transplant
Handa et al. [11]	2006	212	Japan	Japanese	Prospective	TTE	NA	5.7	
Bourdonnais et al. [12]	2008	162	USA	A-A (88%)	Prospective	TTE ± RHC	22/25 (88%)	14	
Baughman et al. [13]	2010	130	USA	50.8% Caucasian	Retrospective	RHC	50/70 (71%)	38.5	Patients with persistent dyspnea
Alhamad et al. [14]	2010	96	Saudi Arabia	NA	Retrospective	TTE	NA	20.8	
Nardi et al. [15]	2011	58	France	65% Caucasian, 31% black	Retrospective	TTE	NA	26	Stage 4 sarcoidosis
Rapti et al. [16]	2013	313	Greece	NA	Cross-sectional	TTE ± RHC	NA	2.9	
Patel et al. [17]	2018	609051	USA	NA	Cohort database	NA	NA	8.7	Healthcare database
Kirkil et al. [2]	2018	452	USA	69% Caucasian, 30 A-A	Retrospective	RHC	29	6.4	
Tiosano et al. [18]	2019	3993	Israël	NA	Cohort database	NA	NA	6.74	Healthcare database
Frank et al. [19]	2019	9106	Germany	NA	Cohort database	NA	NA	2.8	Healthcare database
Huitema et al. [20]	2019	399	Netherlands	Dutch	Prospective	TTE ± RHC	NA	2.9	
Pabst et al. [12]	2020	111	Germany	NA	Prospective	TTE ± RHC	4/5 (80%)	3.6	

Abbreviations: TTE: transthoracic echocardiography; RHC: right heart catheterization NA: not available; USA: United States of America; A-A: African American.

**Table 2:** Main studies on PAH-targeted therapy for SAPH.

Number of Patients	Drug	Results	Study
Retrospective case series including more than 10 patients			
n = 12	Sildenafil	After 4–6 months: improvement in hemodynamics and no change in 6MWTD	[94]
n = 22	Initial monotherapy - Bosentan (n = 12) - Sildenafil (n = 9) - Epoprostenol (n = 1) Combination therapy if inadequate response (n = 8)	After 11–15.2 months: improvement in hemodynamics and in 6MWTD; improvement of NYHA functional class in nine patients	[95]
n = 33	- Sildenafil (n = 29) - Sildenafil + Bosentan (n = 4)	After 6 months: Increase in 6MWTD, BNP levels and TAPSE; improvement of WHO functional class in 14 patients	[96]
n = 13	Prostanoids as monotherapy or in combination therapy - Epoprostenol (n = 7) - Treprostinil (n = 6)	After a mean of 12.7 months: improvement in PVR but not in mPAP At 3 years, improvement in NT-pro BNP levels and WHO functional class	[97]
n = 12	Epropostenol (n = 12) + Tadalafil (n = 4) + Sildenafil (n = 1) + Ambrisentan (n = 1)	After a mean of 4.1 years: improvement in hemodynamics	[98]
n = 97 with severe PH	Monotherapy (n = 83) - ERA (n = 60) - PDE-5i (n = 20) - Epoprostenol (n = 2) - Inhaled Iloprost (n = 1) Combination therapy (n = 14) - ERA + PDE-5i (n = 12) - ERA + Prostanoid (n = 2)	After a median of 4.5 months: improvement in hemodynamics, and NYHA functional class; no change in 6MWTD	[41]
Prospective open-label trial			
n = 15/22 completed trial	- Inhaled Iloprost	After 16 weeks: 8/15 responders (either increased 6MWTD ≥ 30 m or decreased PVR ≥ 20%); overall significant improvement in SGRQ score	[89]
n = 10/21 completed trial	- Ambrisentan	After 24 weeks: improvement in WHO functional class and SGRQ; no change in 6MWTD, BNP levels, Borg scale or SF-36 score	[90]
n = 7/12 completed trial	- Tadalafil	After 24 weeks: no change in 6MWTD, dyspnea, BNP levels or QOL scores	[91]
Randomized placebo-controlled trial			
23/25 completed trial	- Bosentan vs. placebo	After 16 weeks: improvement in hemodynamics compared to placebo; no change in 6MWTD, dyspnea or QOL scores	[92]
8/8 completed trial	- Riociguat vs. placebo	At 1 year: delayed time to clinical worsening compared to placebo (defined as time to all-cause mortality, need for hospitalization because of worsening cardiopulmonary status, >50 m decrease in 6MWTD, or worsening of WHO functional class); improvement in 6MWTD compared to placebo; no change in QOL scores	[93]

Abbreviations: PAH: pulmonary arterial hypertension, SAPH: sarcoidosis-associated pulmonary hypertension, 6MWTD: six-minute walk test distance, mPAP: mean pulmonary arterial pressure, PVR: pulmonary vascular resistance, NYHA: New York Heart Association, WHO: World Health Organization, TAPSE: tricuspid annular plane systolic excursion, BNP: bone natriuretic peptide, ERA: endothelin-1 receptor antagonist, PDE-5i: phosphodiesterase-5 inhibitors, SGRQ: St Georges respiratory questionnaire; QOL: quality of life, SF-36: short-form 36 questionnaire, FAS: fatigue assessment scale.

## Data Availability

Not applicable.

## Abbreviations

6MWD	6 min walk test distance
6MWT	6 min walk test
AA	ascending aorta
CMR	cardiac MRI
CT	computed tomography
CTEPH	chronic thromboembolic pulmonary hypertension
DECT	dual-energy CT
DLCO	lung diffusing capacity for carbon monoxide
ERA	endothelin receptor antagonist
ESC/ERS	European Society of Cardiology/European Respiratory Society
FEV1	forced expiratory volume in 1 s
FVC	forced vital capacity
ILD	interstitial lung disease
LTx	lung transplantation
LV	left ventricle
MRI	magnetic resonance imaging
NYHA	New York Health Organization
OSAS	obstructive sleep apnea syndrome
PA	pulmonary arteries
PAH	pulmonary arterial hypertension
PAWP	pulmonary artery wedge pressure
PDE-5i	phosphodiesterase-5 inhibitors
PFT	pulmonary function testing
PH	pulmonary hypertension
PVR	pulmonary vascular resistance
QOL	quality of life
RHC	right heart catheterization
RPCT	randomized placebo-controlled trial
RVSP	right ventricular systolic pressure
SAPH	sarcoidosis-associated pulmonary hypertension
TRV	tricuspid regurgitant velocity
TTE	transthoracic echocardiography
V/Q	ventilation/perfusion
WASOG	World Association of Sarcoidosis and other Granulomatosis
WHO	World Health Organization
WU	Wood’s units
